# Air and Exercise

**Published:** 1854-02

**Authors:** 


### AIR AND EXERCISE.

No remedy known to men has such a powerful and permanent influence in maintaining or regaining health as the judicious employment of cheerful, exertive exercise in the open air, and if properly attended to in a timely manner, it will cure a large majority of all curable diseases, and will sometimes succeed, when medicines have lost their power.

If you have actual consumption, or are merely threatened with it; or if, from some of your relatives having died with it, you have unpleasant apprehensions of its lurking in your own body; or whether from a diseased liver or disordered stomach, or a dyspeptic condition of the system, the foundations of the dreadful disease are being laid in your own person; or whether by exposure, by over bodily exertion or mental labor, or wasting cares for the present, or anxieties for the future, or by hugging sharp-pointed memories of the past, or by intemperate living, in eating or drinking, or by unwise habits or practices in life, you have originated in your own person the ordinary precursors of consumption, such as hacking cough, pains in the breast, chilliness, wasting of flesh and strength, shortness of breath on exercise—under all these circumstances, a proper attention to air and exercise are indispensable aids—are among the principal, essential means of cure, and are never to be dispensed with; confinement to the regulated temperature of a room in any latitude, is certain death, if persevered in; and if from any cause this air and exercise are not practicable to you, except to a limited extent, it is your misfortune; your not being able to employ them, does not make them the less necessary, *and they have no substitutes.* (See page 78 of "Bronchitis and Kindred Diseases," by W. W. Hall, eighth edition, 1854, Redfield, publisher, 110 and 112 Nassau street, New York.)

When the body is diseased, it is because it is full of diseased, decaying, dead and useless particles; the object of exercise, as well as medicine, is to throw off these particles; medicine does it more quickly, but exercise more safely and certainly, *if there is time* to wait for its effects. Every motion of the body, every bend of the arm, every crook of the finger, every feeling, every breath, every thought, is at the expense, the consumption, the throwing off, of a greater or less proportion of the material body; all muscular motion implies friction, and where there is friction there must be loss. In proportion then as you exercise, you get rid of the old, useless or diseased particles of the body, and by eating substantial, plain, nourishing food, you supply new, healthful, life-giving particles in their stead; therefore every step you take tends to your restoration, provided that step be not taken in weariness or fatigue; for then it prepares the way for a greater destruction of living particles, rather than a removal of the old. You will never fail to find, that whenever you *overdo yourself*, in the way of exercise, you will always feel the worse after it. The exercise must be always adapted to the strength, and the rule is imperative under all circumstances, STOP SHORT OF FATIGUE. This applies to mental as well as to bodily operations. But if you say, as many others have said, and died, "I can't help it," then you must take the consequences and responsibility. If you do not use the means of health, you cannot be cured. If you really and truly *cannot* use them, that inability does not alter the necessity of their observance nor the effect of their neglect.

Take, if possible, an hour's active, cheerful, willing walk, thrice a day; this is many times better than three hours' continuous exercise. The noon walk should be before dinner. If you walk, or leave the house, before breakfast, eat first a cracker or crust of bread. Avoid, during warm weather, in the south and west, and in level or damp situations, the out-door air, including the hour about sunrise and sunset. There is no danger usually, even to invalids, in exercising in the night air, if it be *sufficiently vigorous to keep off a feeling of chilliness*. This should be the rule in all forms of out-door exercise, and is an infallible preventive, as far as my experience extends, against taking cold in any and all weathers, provided it be not continued to over exhaustion or decided fatigue. Such exercise never can give a cold, whether in rain, or sleet or snow, unless there be some great peculiarity in the constitution. It is the conduct *after* exercise which gives the cold; it is the getting cool too quick, by standing or sitting still in a draft of air or open window or cold room. The only precaution needed is, to end the exercise in a room or temperature uncomfortably warm when first entered, and there remain until rested, and no moisture is observed on the surface. (p. 317.)

If working or walking cause actual fatigue, then horseback exercise is the next best for both sexes, but if not able, then ride in a close carriage, especially in cold weather, or when there is a damp raw wind blowing. You may, in the bitterest, coldest weather, secure for yourself the most favorable of all circumstances for recovery—that is, a cool, dry, still atmosphere, by riding several hours a day in a close carriage, well and warmly clad, with your feet on bottles of hot water. The atmosphere of the carriage will not become impure but to a slight extent, as the cold fresh air is constantly coming in at every crevice at the sides and below, while the warm, used air rises to the top, and is expelled by the more powerful currents from without.

It is a laborious business to spend hours every day in exercising, for the mere sake of the exercise; therefore, if possible, devise means of employment which will combine utility with your exercise. The reader's ingenuity may devise methods of accomplishing this, adapted to his condition, and the circumstances by which he is surrounded. Some trim, or bud, or graft fruit-trees, work in a garden, cultivate the vine, or flowers, or plough in fields free of stumps and stones, thus requiring no great effort, yet a steady one, which can be left off at any moment, and followed more or less energetically, so as to produce a very moderate degree of perspiration on the forehead, without fatigue; others saw wood, visit the poor and unfortunate, drive cattle, collect accounts, obtain subscriptions, sell books, distribute tracts, ride on agencies. The great object is, useful, agreeable, profitable employment, in the open air, for several hours every day, rain or shine, hot or cold; and whoever has the determination and energy sufficient to accomplish this, will seldom fail to delight himself and his friends with speedy, permanent and most encouraging results; and be assured, that these alone are the persons who do or can rationally expect to succeed in effectually and permanently warding off the disease when seriously threatened, or in arresting its progress permanently, when wholly unexpected, by themselves, their friends, or their physicians.

While exercise is important in working off the old, useless, decayed, dead particles from the system, it is equally advantageous in keeping the body warm, by driving the blood to the skin, and keeping it soft and moist; for persons who have a dry, harsh, cold skin, are never well. But pure air is as important as exercise, because the food we eat never becomes blood, until it meets in the lungs the air we breathe; if then we do not take in enough air, or what we do take in is impure, the blood will be imperfect and impure, and, in proportion, unfit to nourish, strengthen and vivify the body. And as in threatened consumption the lungs work more or less imperfectly, and consume less air than the system requires, so much the more need that the air which is consumed should be of the purest kind possible. Therefore, every hour spent out of doors in the pure air, *fatigue and chilliness being absent*, adds that much to the certainty of your recovery. Thus you see that, while exercise works the old diseased particles from your body, pure air puts the finishing stroke of perfection to the new particles which are to take their place, and the whole body, in proportion, becomes new and fresh, and healthful and young. And whatever advice is given you in other printed or written papers, it is designed as an *aid* to bring about these things in a shorter time and easier way. This *aid* is needed in most cases, because, unfortunately, the disease has been neglected or mistreated so long, that nature has lost the power, to a great extent, of helping herself, and medicine must be taken, or the patient perish.

There are two dangers in taking exercise, that of overdoing it, and of getting cool too quick afterwards. Therefore observe the following rules:

If you ride and walk on any one occasion, do the riding first, then the walk will warm you up; but riding after a walk, you get chilled before you know it.

At the end of a ride or walk, do not, for a single moment, sit or stand still anywhere out of doors, nor on damp places, nor on stone or iron seats. Never end a walk or ride in a new building, or in a room which has been closed for some days, or has no fire in it, especially in winter. Walk quickly, cheerfully, with the chin on or above a horizontal line. Make no other effort to walk straight, except thus to elevate your chin. In other words, hold up your head. Breathe habitually with your mouth closed, in damp or cold weather; and in going into the out-door air, close it before you leave the house, and keep it closed until you get warm, especially after speaking or singing.

Embrace every opportunity of running up a pair of stairs, or up a hill, with the lips closed; a dozen times a day, if possible. A rapid run of fifty or a hundred yards and back, three or four times a day, with the mouth closed, will be of inestimable advantage. The reasons you can study out at your leisure.

But simple as these things are, never attempt them without the special advice of an experienced physician, for in certain forms of heart affections, as every practitioner well knows, as also in one or two other ailments, such exercises would, in some cases, cause certain and speedy death.

It is of high importance to the healthy who wish to keep so, and to the sick who are in search of so great a happiness as that of being sound and well again, to breathe habitually with the lips closed in cold weather, in going from a warmer to a cooler, or from a cooler to a warmer atmosphere, the injury is perhaps equally great either way. Close the mouth *before leaving* a concert room, or church, or other warm apartment, and keep it resolutely closed until you have walked far and fast enough to have hastened the circulation of the blood, and made it more full, as well as active.

In going into a warm apartment, from the cold out-door air, the same direction is of not less importance; nor should you go at once to the fire; a delay of two or three minutes is sufficient in this case. The object, in both cases, is the same, to prevent a sudden transition from heat to cold, or the contrary. Such sudden transitions give pain to the solid tooth, or discomfort, when made to a single square inch of the skin; and when it is remembered that the air passages are among the most delicate structures of the body, and that the lungs, if spread out on a wall, would cover a surface ten times larger than the whole skin would do, the importance of the subject must strongly impress every reflecting mind.

With the above precaution, you need not be afraid of out-door air, night or day, as *long as you are in motion sufficient to keep off a feeling of chilliness;* hence, in cold weather, exercise on foot is preferable to riding. While walking in moderately cold weather, the hands should be covered with a thin pair of gloves, such as silk or thread, and woolen ones in mid-winter. If you have to ride in winter, endeavor to have clothing enough to prevent a *feeling of chilliness,* but be careful to wear a *loose fitting* boot or shoe; never put on a new pair, winter or summer, when starting on a journey, or coming to the city. In very cold or windy weather, ride in a close carriage.

